# Precocious puberty in Turner Syndrome: report of a case and review of the literature

**DOI:** 10.1186/1824-7288-38-54

**Published:** 2012-10-17

**Authors:** Nicola Improda, Martina Rezzuto, Sara Alfano, Giancarlo Parenti, Pietro Vajro, Claudio Pignata, Mariacarolina Salerno

**Affiliations:** 1 Department of Pediatrics, Federico II University of Naples, Naples, Italy; 2 Chair of Pediatrics, University of Salerno, Salerno, Italy

**Keywords:** Turner syndrome, Precocious puberty, GnRH analog therapy

## Abstract

**Introduction:**

Turner Syndrome (TS) is caused by monosomy or structural abnormalities of the X chromosome, with a prevalence of about 1/2000 females live birth. Most important clinical features of TS are short stature and gonadal failure. Approximately one third of girls with TS may undergo spontaneous puberty. Here we report on the case of a girl with a rare 45X0/47XXX mosaic TS exhibiting a precocious puberty.

**Case report:**

The patient was diagnosed with TS at the age of 4 years, upon a diagnostic work-up for dysmorphic features. Chromosome analysis revealed a mosaic karyotype (45X0/47XXX). She presented with normal height and normal growth velocity so that Growth Hormone (GH) therapy was not started. She was referred to our Department at the age of 7 years and 10 months, because of vaginal bleeding. A physical examination revealed a Tanner stage III for breast and Tanner stage III for pubic hair development. Height and weight were within the normal range for age. Psychological evaluation showed moderate global developmental delay, together with emotional and social immaturity and reading difficulties. The growth rate was accelerated. Her bone age was 10 years. Pelvic ultrasound demonstrated increased size for age of both the uterus and the ovaries, with bilateral ovarian follicles. GnRH stimulation test revealed pubertal response of gonadotropins (peak LH 22.5 mIU/ml). MRI of the brain was normal. These clinical, radiologic and laboratory findings were consistent with a diagnosis of idiopathic central precocious puberty; therefore, GnRH analog therapy was started, in order to slow pubertal progression and to preserve adult stature. Furthermore, GH treatment was added to further improve adult height.

**Conclusion:**

Our case highlights the possibility of precocious puberty as an atypical clinical feature of TS. Thus, precocious puberty may occur in TS girls when a dosage compensation by the cell line with more than two X chromosomes allows normal ovarian function. GnRH analog therapy in addition to GH treatment should be recommended in TS girls with precocious puberty in order to slow pubertal progression and to preserve adult stature.

## Background

Turner syndrome (TS) is a relatively common chromosomal disorder caused by complete or partial X monosomy, with a prevalence of approximately 1/2000 females live births [1]. The genetic background of TS is highly variable. The most frequent occurring karyotypes are 45,X, karyotypes with an isochromosome of X (i(Xq) or i(Xp)), the mosaic karyotype of 45,X/46XX, and karyotypes containing an entire Y chromosome or parts thereof [2]. The mosaic TS karyotype occurs in approximately 30% of all patients with TS [3]. Short stature, gonadal dysgenesis and congenital malformations are the main clinical features, although a number of others signs and symptoms are seen more frequently with the syndrome [1]. Although in TS there is not a clear genotype/phenotype correlation, girls with mosaic TS who have a normal cell line, or an extra X chromosome tend to exhibit milder phenotypes [3].

Absent pubertal development and primary amenorrhea occurs in most individuals with TS, due to accelerated loss of oocytes in the 45,X ovary, leaving few follicles in a fibrous strike by birth. Approximately one third of girls with TS undergo spontaneous puberty, but only half of those complete puberty with menarche. Spontaneous pregnancies are rare (2-5%) [4]. A few rare cases of precocious puberty have been described, mainly in girls with mosaic TS (45X0/46XX or X structural abnormalities) [5-8].

We report on the first case of precocious puberty in a 45X0/47XXX mosaic TS.

## Case report

A female child was born pre-term (32 weeks of gestation) with a birth weight of 2.900 kg and a length of 49 cm. Postnatal period was uneventful. She was diagnosed with TS at the age of 4 years, upon a diagnostic work-up for dysmorphic features. High resolution chromosome analysis revealed a mosaic karyotype (55% 45X0/45% 47XXX); FISH analysis excluded Y-chromosome-specific sequences. Her stature was normal and within the target height, no other associated abnormalities were detected, thus she was followed by the general pediatrician.

**Figure 1:**
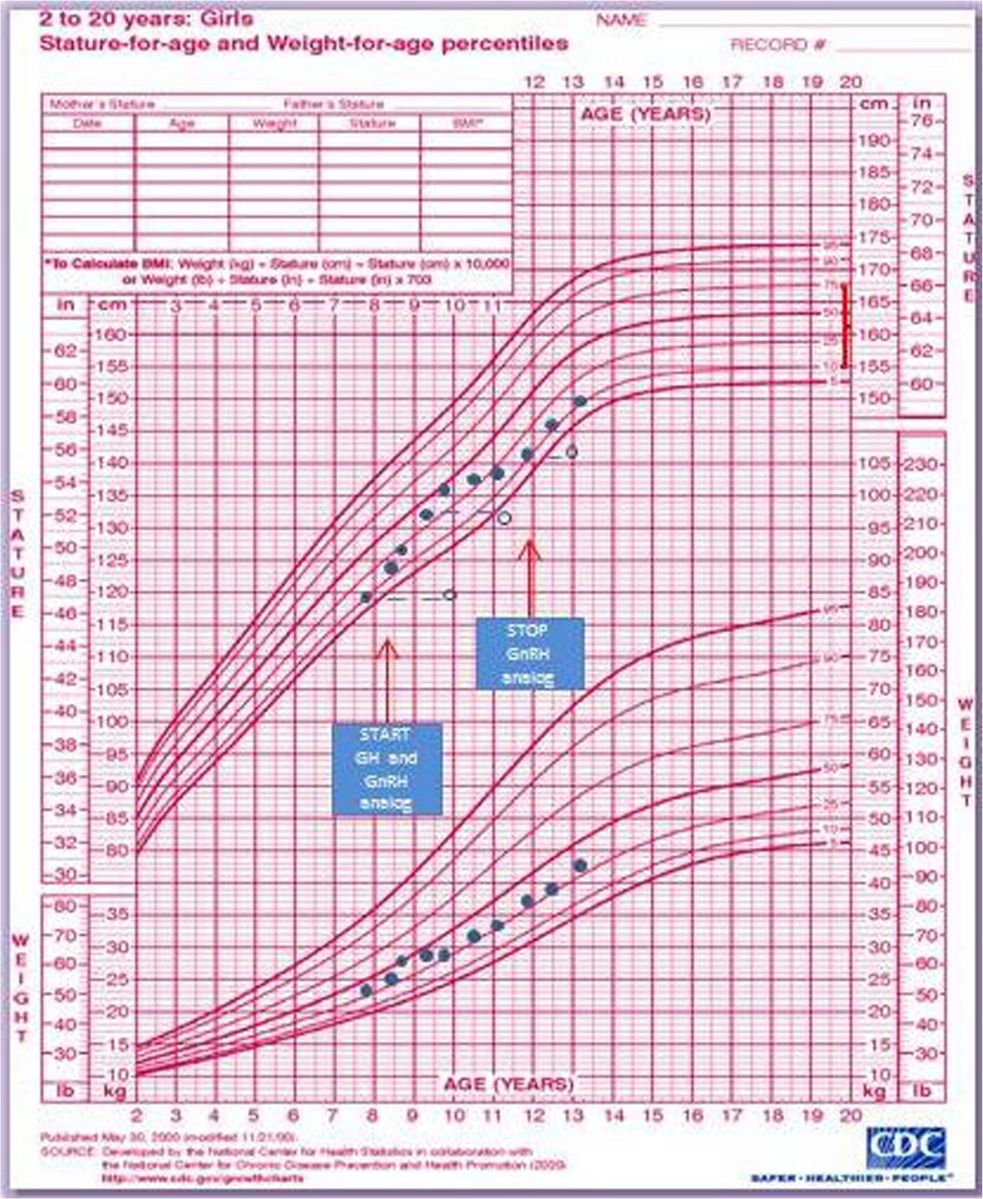
Growth chart of our patient.

She was referred to our Department at the age of 7 years and 10 months, because of vaginal bleeding. A physical examination revealed a Tanner stage III for breast development, Tanner stage III for pubic hair development, axillary hair and scoliosis. Height and weight were within the normal range for age (height 126 cm, 25th percentile, and weight 26 kg, 25-50th percentile, respectively). The growth rate was accelerated (12 cm/year) (Figure 1). Psychological evaluation showed moderate global developmental delay, together with emotional and social immaturity and reading difficulties. Skeletal maturation, evaluated by a left wrist x-ray was 10 years. Pelvic ultrasound demonstrated increased size for age of both the uterus (longitudinal diameter 53mm) and the ovaries (right ovary volume 3 ml and left 3.3 ml) with bilateral ovarian follicles. Abdominal and cardiac ultrasound, as well as routine hematological and biochemical analysis were normal. Endocrine evaluation revealed FSH levels 7.66 mIU/mL, LH levels 1.68 mIU/mL, and slightly elevated estradiol levels (28.2 pg/mL). GnRH stimulation test revealed pubertal response of gonadotropins with LH peak value of 22.5 mIU/ml and FSH peak value of 17.8 mIU/mL. Furthermore, assessment of thyroid function showed thyroid stimulating hormone (TSH) 5.5 μΙU/mL (normal range: 0.3-4.2 μΙU/mL), with normal levels of free T4 (1.25 ng/dl, normal range 0.8-1.7), and positive thyroid anti-TPO autoantibodies (100 IU/l, normal value less than 35), thus suggesting a condition of mild subclinical hypothyroidism in the context of autoimmune thyroiditis [9-11]. Other autoimmune diseases were ruled out by appropriate investigations [12-15]. Serum IGF-1 level was within normal limits. MRI of the brain was normal.

These clinical, radiologic and laboratory findings were consistent with a diagnosis of idiopathic central precocious puberty; therefore, GnRH analog therapy was started, in order to slow pubertal progression and to preserve maximum adult stature [5,16]. Furthermore, GH treatment was added to further improve adult height.

At the chronological age of 12 years and bone age of 13 years, GnRH analog therapy was discontinued, resulting in recovery of pubertal development which was completed by menarche at the age of 12 yr and 10 months. She is now 13 years old, she is still receiving GH treatment, her near final height is 150 cm, slightly below the target height (Figure 1).

## Discussion

We report on the first case of precocious puberty in a TS with a rare 45X0/47XXX mosaic karyotype.

TS is associated with a constellation of potential abnormalities involving many organ systems, making it a challenging disorder for health care providers and families. The genetic background of TS is highly variable. About 30% of TS girls have a mosaic chromosomal structure, with only 1% showing a 45X0/47XXX mosaicism [17]. The correlation between genotype and phenotype is not yet well understood, but generally patients with a 45,X karyotype tend to have a more severe phenotype than those who are mosaic with a normal cell line [2].

Short stature, ovarian dysgenesis and infertility are clinical hallmarks in the majority of patients with TS. The incidence of spontaneous puberty in TS is reported to be about one third. In an Italian retrospective multicenter study, 33.5% of 522 patients older than 12 yr with Turner syndrome presented spontaneous pubertal development, but only 16.1% completed puberty with menarche [18].

Precocious puberty in TS patients is very rare. To date, only five cases have been reported in the Literature, four of them showing mosaic TS and one a karyotype with structural abnormality of one X chromosome [5-8].

To our knowledge this is the first case of precocious puberty reported in a TS with a mosaic karyotype characterized by a cell line with X chromosome monosomy and a cell line with three copy of the X chromosome, thus suggesting a post-zygotic unbalanced disjunction of the sex chromosomes. In addition to precocious puberty, our patient showed other peculiar clinical features, such as normal height and neuro-psychological problems, likely related to the quantitative proportion of 45X to 47XXX cell-lines in different tissues and organs [19].

Triple X syndrome (47XXX) is a sex chromosome aneuploidy that occurs in approximately 1 in 1000 female births. Although non-mosaic 47,XXX karyotypes are the most frequent, mosaicism occurs in approximately 10% of cases and in many combinations such as 46XX/47XXX or 47XXX/48XXXX, or in combination including Turner syndrome cell lines such as 45X/47XXX or 45X/46XX/47XXX. Clinical characteristics include epicanthal folds, hypertelorism, upslanting palpebral fissures, clinodactyly, overlapping digits, pes planus and pectus excavatum. The majority of cases are asymptomatic with normal pubertal onset and sexual development [20]. However, pubertal abnormalities have been reported in Triple X syndrome, ranging from premature ovarian insufficiency [21] to precocious puberty [22,23].

Genes located on the proximal region of the short arm of the X chromosome are important for normal ovarian function and development and the haploinsufficiency of these genes is thought to be implicated in the pathogenesis of gonadal dysgenesis associated with TS [24]. On the contrary, spontaneous puberty has been reported with a significantly higher frequency among mosaic TS with cell lines having more than one X. These observations suggest a cardinal influence of the X chromosome on the appearance of spontaneous puberty [18]. In keeping with this, our case suggests that even precocious puberty may occur in TS girls when a dosage compensation by the cell line with more than two X chromosomes allows normal ovarian function.

The pathogenetic mechanism for central precocious puberty in sex chromosome aneuploidies, particularly in TS, is still unclear. Abnormalities in the hypothalamic feedback system, with increased levels of gonadotropins to compensate for blunted ovarian function, or FSH surge commonly seen in TS before the ovarian failure have been hypothesized [7]. Elevated levels of TSH have also been proposed as a mechanism in causing precocious puberty in girls with TS, due to the interaction between TSH and human FSH receptor [25]. In our patient it seems unlikely that the slightly increase in TSH levels may be responsible for precocious puberty, because TSH levels were stable over time and no other functional abnormalities were detected.

The decision to treat with GnRH analog a condition such as TS, generally associated with ovarian failure, is challenging. The observation that untreated precocious puberty is generally associated with impaired adult height due to bone age advancement and that short stature is *per se* a hallmark of TS prompted us to undertake a treatment with GnRH analog [26]. Again, despite a normal GH secretion following a pharmacological stimulation test [27], evidence indicates that a GH treatment may improve adult height in TS as well as in other syndromes [28,29]. For this reason, the initiation of GH therapy is currently considered as soon as growth failure is evidenced even though short stature is not present [4]. In our patient, GH therapy was not initially started due to normal height and normal growth velocity. However, after the diagnosis of precocious puberty, GH therapy was considered in order to improve adult height.

## Conclusion

Our case highlights the possibility of precocious puberty as an atypical clinical feature of TS. Precocious puberty may occur in TS girls when a dosage compensation by the cell line with more than two X chromosomes allows normal ovarian function. GnRH analog therapy in addition to GH treatment should be recommended in TS girls with precocious puberty in order to slow pubertal progression and to preserve adult stature.

## Consent

Written informed consent was obtained from the parents of the patient for publication of this Case report and any accompanying images. A copy of the written consent is available for review by the Editor-in-Chief of this journal.

## Abbreviations

TS: Turner Syndrome; GH: Growth Hormone.

## Competing interests

The authors declare that they have no competing interests.

## Authors' contributions

All authors have equally participated in drafting of the manuscript and/or critical revision of the manuscript for important intellectual content. All authors read and approved the final manuscript.
